# Elevated pre-pregnancy testosterone is associated with gestational diabetes mellitus: an observational cohort study

**DOI:** 10.3389/fendo.2025.1618107

**Published:** 2025-10-16

**Authors:** Xufan Luo, Meng Li, Yan Long, Chunmei Ying, Chaoyan Yue

**Affiliations:** ^1^ Obstetrics & Gynecology Hospital of Fudan University, Shanghai Key Lab of Reproduction and Development, Shanghai Key Lab of Female Reproductive Endocrine Related Diseases, Shanghai, China; ^2^ Obstetrics and Gynecology Hospital of Fudan University, Shanghai, China

**Keywords:** pre-pregnancy testosterone, gestational diabetes mellitus, body mass index, odds ratio, age

## Abstract

**Context:**

Pre-pregnancy testosterone increase may be closely related to the occurrence of gestational diabetes mellitus (GDM).

**Objective:**

Our aim is to explain the relationship between pre-pregnancy testosterone levels and the risk of GDM and provide evidence for early clinical warning.

**Methods:**

We conducted a retrospective cohort study on 4174 parturient. The exposure factor is pre-pregnancy testosterone, with the primary outcome was GDM (A1+A2) and the secondary outcome was GDM A2. We used trend testing, multivariate logistic regression models, smooth curve fitting, and age and BMI for subgroup analysis and interaction analysis to evaluate the relationship and odds ratio between different pre-pregnancy testosterone levels and GDM risk.

**Results:**

Elevated levels of pre-pregnancy testosterone are closely related to the onset of GDM Compared with the control group, women with testosterone levels between Q4 nmol/L had an OR value of 1.76 (95% Confidence Interval: 1.38,2.25) for GDM (A1+A2). The OR value for GDM A2 is 2.26 (95% CI: 1.27,4.00). In terms of sensitivity analysis, it was also observed that elevated pre-pregnancy testosterone increased the risk of GDM, especially in the age<35 and BMI<24 groups. Pre-pregnancy testosterone had a greater effect on GDM A2, with OR values of 1.42 (95%CI: 1.04,1.94) and 1.86 (95% CI: 1.30,2.66).

**Conclusion:**

Pre-pregnancy testosterone testing is associated with the risk of developing GDM, and women age<35 or BMI<24 need to pay more attention to pre-pregnancy testosterone. Pre-pregnancy testosterone can serve as a potential biomarker for risk stratification of GDM.

## 1 Introduction

Gestational diabetes mellitus (GDM) is a common complication of pregnancy. The series of complications that this can cause, such as intrauterine growth restriction, have become a major challenge for pregnant (1–4). The global incidence rate of GDM is about 14% (5, 6). A meta-analysis shows that the total incidence rate of GDM in China is 14.8% (7).The high-risk factors for the increasing prevalence of GDM include advanced maternal age, pre-pregnancy overweight or obesity (8, 9).

Testosterone is a steroid hormone mainly secreted by women’s ovaries and adrenal glands, which can cause diabetes through insulin resistance, inflammation and oxidative stress (10, 11). Pre-pregnancy testosterone test is a mature item requiring only a single blood draw, which may be a risk related to GDM. However, there is currently limited research on the correlation between pre-pregnancy testosterone and GDM. The existing research is mainly limited to the relationship between testosterone during pregnancy and GDM, and the related research results are controversial. At present, there are many studies indicating that higher testosterone may increase non-pregnancy women’s risk of metabolic diseases, including insulin resistance and diabetes (4, 12–17). Meanwhile, some studies have explained the relationship between elevated testosterone during pregnancy and GDM (18, 19), but there is relatively little research on the relationship between pre-pregnancy testosterone and GDM. Contrary studies have shown that no increase in testosterone levels has been observed in women with gestational diabetes (12).

To explore the relationship between pre-pregnancy testosterone level and gestational diabetes is of great significance for pregnant women’s health management and fetal growth and development (15). Our retrospective cohort study included 4174 singleton mothers and used odds ratios (OR) and other indicators to describe the relationship between pre-pregnancy testosterone levels and GDM (20). Trend testing is used to obtain the relationship between different pre-pregnancy testosterone levels and the risk of GDM. We also conducted subgroup interaction analysis between BMI and age and GDM (A1+A2) and GDM A2, respectively. Finally, we also used smooth curve fitting to intuitively obtain pre-pregnancy testosterone, BMI, The relationship between age and GDM risk.

## 2 Materials and methods

### 2.1 Design and participants

The detection time of pre-pregnancy testosterone is from January 2018 to September 2023.For these women with baseline testosterone data, whether they developed GDM in the following two years of pregnancy was taken as the observation outcome. Therefore, this study included a total of 4174 pregnant women. When pregnant women visit the clinic between 8 and 12 weeks of pregnancy, they are included in the queue. The exclusion criteria for the study are as follows: threatened miscarriage or stillbirth, more than 24 weeks at the first outpatient visit, pregnancy complicated with endocrine disorders, pregnancy complicated with respiratory system diseases, pregnancy complicated with urinary system diseases, pregnancy complicated with circulatory system diseases, and no testosterone test conducted before 2 years of confirmed pregnancy. Approval was obtained from the Research Ethics Committee of the Obstetrics and Gynecology Hospital of Fudan University (approval number: 2024-189).

### 2.2 Variables and measurements

Pre-pregnancy testosterone testing is performed using a fully automated chemiluminescence analyzer (Beckman Coulter Dxl800, original reagent) to analyze testosterone levels. The confounding factors for adjustment include BMI; Family history of hypertension; Family history of diabetes; Tobacco; Alcohol; IVF; ALT; Cr; TCH; TG; Gestational weeks; GLU; Education. Pregnancy week, weight, education level and other information are obtained through the records in HIS.

### 2.3 Outcome and measurements

The primary outcome measure is GDM (A1+A2). GDM (A1+A2) is defined as all types of GDM. During 24–28 weeks of pregnancy, the oral glucose tolerance screening test recommended by the International Association of diabetes and Pregnancy Research Groups was used to test the pregnant women, that is, the 75g glucose tolerance test. GDM was diagnosed when the fasting blood glucose ≥5.1mmol/L or 1-hour glucose ≥10mmol/L or 2-hour glucose ≥ 8.5mmol/L. The secondary outcome is GDM A2, which is defined as GDM requiring drug control. Through dietary control, fasting blood glucose ≥5.8mmol/L and 2-hour blood glucose ≥6.7mmol/L requires insulin to control blood glucose.

### 2.4 Statistical analysis

For continuous variables, data is expressed as mean (SD), while for binary variables, data is expressed as percentage (%). For quantitative data, analysis of variance is used for testing, and for categorical data, chi square test is used. When *P* < 0.05, statistical differences are considered. We created a smooth curve fitting graph of pre-pregnancy testosterone, age, and BMI to examine their relationship with GDM. Differentiate the pre-pregnancy testosterone values into four intervals based on quartiles, use a logistic regression model to test and calculate the adjusted odds ratio (OR) for each interval, and conduct a trend test. The calculation of OR value is achieved through formulas. $OR=\frac{a*d}{b*c}$ .

a:Number of pregnant women with elevated pre-pregnancy testosterone and GDM. b:Number of pregnant women with elevated pre-pregnancy and without GDM. c: Number of pregnant women with GDM in the reference group. d:Number of pregnant women without GDM in the reference group.

Finally, subgroup analysis was conducted on age and BMI, and interaction tests were performed to compare their respective effects on the onset of GDM. *P* < 0.05 is considered statistically significant. All reported P-values are two-sided. Statistical analyses were performed using R version 3.5.1 (R Foundation for Statistical Computing) and the software IBM SPSS (version 21.0. IBM; Armonk, NY).

## 3 Results

**Figure 1:**
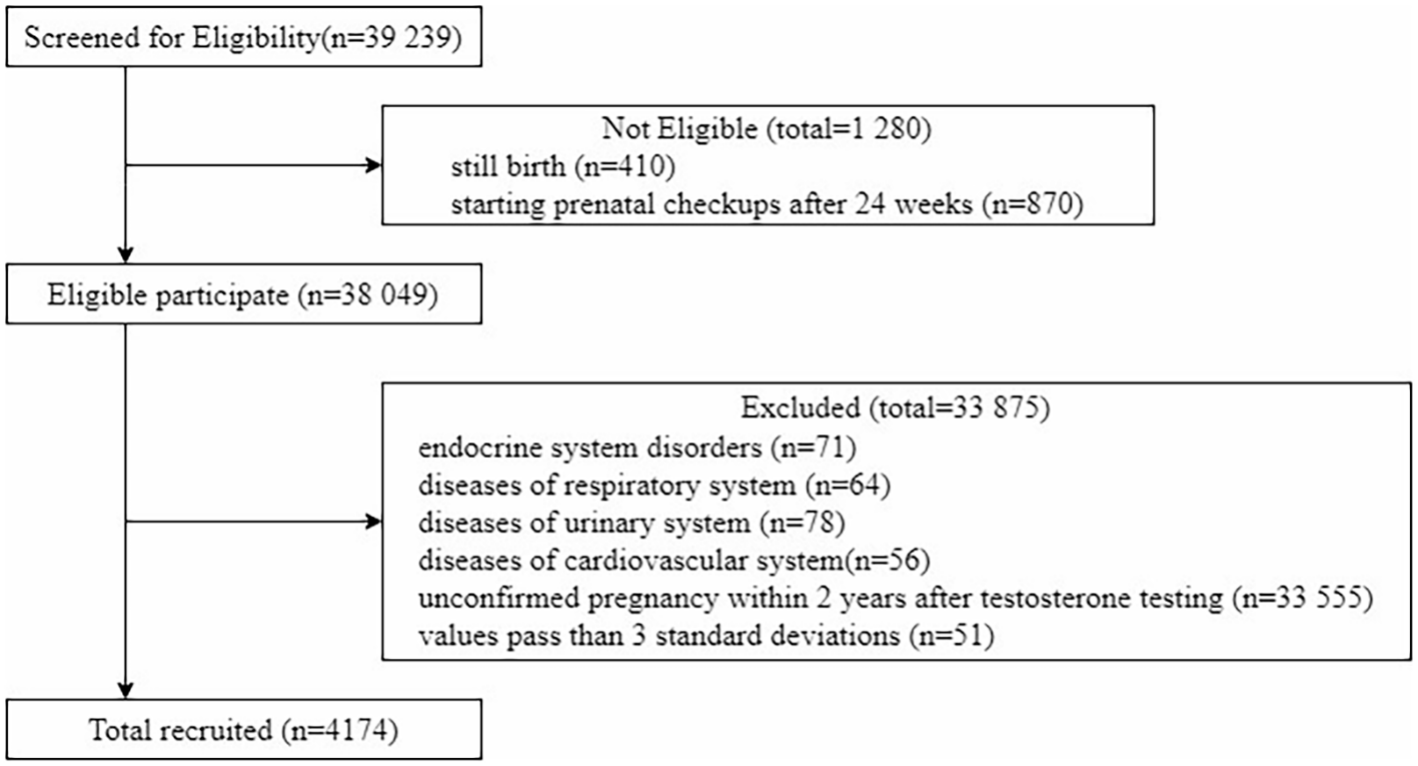
Flowchart of participants.

According to a series of inclusion and exclusion criteria, a total of 4174 pregnant women were included in the study out of 39239 pregnant women. A total of 771(18.47%) pregnant women were diagnosed with GDM(A1+A2), of which 136 were diagnosed with GDM A2 type (**Figure 1**). Based on literature review, this study divided pre-pregnancy testosterone into four quartiles: Q1 (0.01-1.24nmol/L), Q2 (1.25-1.63 nmol/L), Q3 (1.64-2.08 nmol/L), and Q4 (2.09-3.97 nmol/L).

The baseline characteristics may be related to pre-pregnancy testosterone levels and the occurrence of GDM(A1+A2), as described in detail. Except for pre-pregnancy testosterone, all other data were measured or obtained during pregnancy (**Table 1**).

**Table 1:** Baseline characteristics.

Characteristic	Testosterone quartile				
	Q1	Q2	Q3	Q4	*P*
No. of participants	1042	1028	1031	1073	
Pre-pregnancy testosterone(nmol/L)	0.89 ± 0.27	1.44 ± 0.11	1.83 ± 0.13	2.57 ± 0.42	<0.01
Test of gestational weeks	10.20 ± 1.87	10.36 ± 2.25	10.29 ± 2.08	10.46 ± 2.18	0.03
Age(years)	32.65 ± 3.70	31.70 ± 3.90	31.24 ± 3.53	30.80 ± 3.34	<0.01
BMI	21.74 ± 2.98	21.62 ± 3.04	21.83 ± 3.14	22.21 ± 3.52	<0.01
ALT(U/L)	18.41 ± 15.51	19.24 ± 17.50	19.95 ± 18.95	20.24 ± 17.03	0.07
Cr(μmol/L)	43.48 ± 6.07	43.48 ± 5.86	43.93 ± 6.14	43.51 ± 6.12	0.25
Total cholesterol (mmol/L)	4.47 ± 0.81	4.51 ± 0.76	4.53 ± 0.79	4.55 ± 0.77	0.105
Triglyceride (mmol/L)	1.27 ± 0.59	1.33 ± 0.59	1.35 ± 0.63	1.38 ± 0.64	<0.01
GLU (mmol/L)	4.54 ± 0.38	4.49 ± 0.38	4.51 ± 0.43	4.51 ± 0.43	0.04
Family history of hypertension					0.47
No	858 (82.50%)	848 (82.49%)	873 (84.68%)	898 (83.77%)	
Yes	182 (17.50%)	180 (17.51%)	158 (15.32%)	174 (16.23%)	
Family history of diabetes					0.80
No	959 (92.21%)	951 (92.51%)	962 (93.31%)	992 (92.54%)	
Yes	81 (7.79%)	77 (7.49%)	69 (6.69%)	80 (7.46%)	
Tobacco					0.10
No	1030 (98.85%)	1020 (99.22%)	1023 (99.22%)	1054 (98.23%)	
Yes	12 (1.15%)	8 (0.78%)	8 (0.78%)	19 (1.77%)	
Alcohol					0.73
No	1012 (97.12%)	1006 (97.86%)	1006 (97.58%)	1048 (97.67%)	
Yes	30 (2.88%)	22 (2.14%)	25 (2.42%)	25 (2.33%)	
IVF					0.42
No	930 (89.25%)	933 (90.76%)	931 (90.30%)	980 (91.33%)	
Yes	112 (10.75%)	95 (9.24%)	100 (9.70%)	93 (8.67%)	
Education					0.05
Postgraduate	277 (26.92%)	223 (21.99%)	220 (21.72%)	205 (19.21%)	
Bachelor's degree or above	473 (45.97%)	489 (48.22%)	499 (49.26%)	525 (49.20%)	
College	179 (17.40%)	187 (18.44%)	191 (18.85%)	215 (20.15%)	
Senior high school	29 (2.82%)	31 (3.06%)	30 (2.96%)	38 (3.56%)	
Junior high school and below	71 (6.90%)	84 (8.28%)	73 (7.21%)	84 (7.87%)	
GDM (A1+A2)					<0.01
No	865 (83.01%)	863 (83.95%)	848 (82.25%)	827 (77.07%)	
Yes	177 (16.99%)	165 (16.05%)	183 (17.75%)	246 (22.93%)	
GDM A2					0.05
No	719 (96.90%)	692 (95.05%)	706 (95.92%)	746 (94.07%)	
Yes	23 (3.10%)	36 (4.95%)	30 (4.08%)	47 (5.93%)	

TCH, Total cholesterol; TG, triglyceride; ALT, alanine aminotransferase; BMI, body mass index; Cr, creatinine.

The main and secondary outcomes of pre-pregnancy testosterone ORs and adjusted ORs are shown in **Table 2**. Overall, the higher the pre-pregnancy testosterone, the higher the probability of both GDM(A1+A2) and GDM A2. When the pre-pregnancy testosterone is at the Q4 percentile, the odds ratio of GDM(A1+A2) is 1.76 times higher than that of the control group (OR = 1.76,95% CI: 1.38,2.25, *P* < 0.05), and there is a difference in the trend test between the rising trend of pre-pregnancy testosterone and the overall incidence rate of GDM(*P* < 0.05). Similar conclusions have been drawn regarding GDM A2. More noteworthy is that when pre-pregnancy testosterone levels are in Q4, the odds ratio of developing GDM A2 increases by 2.26 times compared to the control group (OR = 2.26,95% CI: 1.27,4.00, *P* < 0.05), which is much higher than other values.

**Table 2:** Individual effect of testosterone on GDM.

Exposure	Non-adjusted		Adjusted	
	OR (95% CI)	*P* value	OR (95% CI)	*P* value
Primary outcome				
GDM (A1+A2)				
Continuous Testosterone	1.28 (1.14,1.44)	<0.05	1.38 (1.22,1.57)	<0.05
Testosterone quartile				
Q1	Reference		Reference	
Q2	0.93 (0.74,1.18)	0.57	1.09 (0.85,1.41)	0.49
Q3	1.05 (0.84,1.32)	0.65	1.26 (0.98,1.62)	0.07
Q4	1.45 (1.17,1.80)	<0.05	1.76 (1.38,2.25)	<0.05
*P* value for trend		<0.05		<0.05
Secondary outcomes				
GDM A2				
Continuous Testosterone	1.38 (1.08,1.76)	<0.05	1.42 (1.08,1.85)	<0.05
Testosterone quartile				
Q1	Reference		Reference	
Q2	1.63 (0.95,2.77)	0.07	1.97 (1.10,3.54)	<0.05
Q3	1.33 (0.76,2.31)	0.31	1.58 (0.86,2.92)	0.14
Q4	1.97 (1.18,3.28)	<0.05	2.26 (1.27,4.00)	<0.05
*P* value for trend		<0.05		<0.05

Adjust for: Age; BMI; Family history of hypertension; Family history of diabetes; Tobacco; Alcohol; IVF; ALT; Cr; TCH; TG; Gestational weeks; GLU; Education.

In terms of sensitivity analysis in **Table 3**, using pre-pregnancy testosterone as an exposure factor, we found that through subgroup analysis of age and BMI, pre-pregnancy testosterone also has certain value in predicting the probability of GDM onset. In terms of age grouping, the ratio of GDM (A1+A2) and GDM A2 of pregnant women age<35 was increased by 35% (OR = 1.35,95% CI:1.17,1.56, *P* < 0.05) and was increased by 42% (OR = 1.42,95% CI: 1.04,1.94, *P* < 0.05), respectively. The incidence rate was higher than that of the control group and had a statistical difference. In pregnant women aged≥35, the ratio of GDM (A1+A2) was increased by 53% (OR = 1.53,95% CI: 1.13,2.07, *P* < 0.05). While there is no statistical difference was observed on the ratio of GDM A2.In terms of BMI, pregnant women with BMI<24, the risk of GDM (A1+A2) increased by 46% (OR = 1.46, 95% CI: 1.26,1.71, *P* < 0.05), and the probability of GDM A2 increased by 86% (OR = 1.86, 95% CI: 1.30,2.66, *P* < 0.05). Among pregnant women with BMI ≥ 24, there was no statistically significant difference in the results. We believe that the reason for the lack of statistical differences between age ≥ 35 and BMI ≥ 24 is that advanced age and high BMI are already high-risk factors for GDM and the influence of pre-pregnancy testosterone is weaker than these two factors.

**Table 3:** Subgroup analysis of the association between age and BMI on GDM.

Outcome	Non-adjusted odds ratio (95% CI)	*P*-value	Crude *P* for interaction	Adjusted odds ratio (95% CI)	*P*-value	Adjust *P* for interaction
Age						
GDM (A1+A2)			0.18			0.45
GDM A2			0.94			0.98
Age<35						
GDM (A1+A2)	1.32 (1.16, 1.51)	<0.05		1.35 (1.17, 1.56)	<0.05	
GDM A2	1.51 (1.13, 2.01)	<0.05		1.42 (1.04, 1.94)	<0.05	
Age≥35						
GDM (A1+A2)	1.62 (1.25,2.10)	<0.05		1.53 (1.13, 2.07)	<0.05	
GDM A2	1.47 (0.91,2.39)	0.12		1.43 (0.81, 2.52)	0.22	
BMI						
GDM (A1+A2)			0.68			0.16
GDM A2			0.18			0.02
BMI <24						
GDM (A1+A2)	1.25 (1.09,1.44)	<0.05		1.46 (1.26, 1.71)	<0.05	
GDM A2	1.53 (1.09,2.14)	<0.05		1.86 (1.30, 2.66)	<0.05	
BMI≥24						
GDM (A1+A2)	1.19 (0.97,1.45)	0.10		1.23 (0.97, 1.56)	0.08	
GDM A2	1.09 (0.76,1.56)	0.64		1.03 (0.69, 1.56)	0.87	

Adjust for: Age; BMI; Family history of hypertension; Family history of diabetes; Tobacco; Alcohol; IVF; ALT; Cr; TCH; TG; Gestational weeks; GLU; Education.

**Figure 2:**
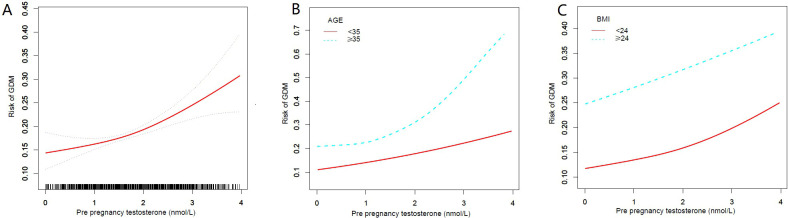
**(A)** The dose-response relationship between pre-pregnancy testosterone and the risk of GDM. **(B)** Dose response relationship between pre pregnancy testosterone and GDM risk grouped by age <35 and ≥35. **(C)** Dose response relationship between pre pregnancy testosterone and GDM risk grouped by BMI <24 and ≥24.

As **Figure 2A** shows, through curve fitting of the risk of pre-pregnancy testosterone and GDM, the results also suggest that with the increase of pre-pregnancy testosterone, the incidence rate of GDM gradually increases. As shown in **Figure 2B**, the increase of pre-pregnancy testosterone is also accompanied by the increase of the incidence of GDM, and when the pre-pregnancy testosterone is greater than 2nmol/L, the incidence rate of GDM in pregnant women aged 35 and above sharply increases and is significantly higher than that in pregnant women under 35. In terms of BMI, as shown in **Figure 2C**, with the increase of pre-pregnancy testosterone, the incidence of GDM gradually increases, and the increase is slightly higher in pregnant women with BMI<24 than in pregnant women with BMI ≥ 24.

## 4 Discussion

### 4.1 Main results

Our study analyzed the relationship between pre-pregnancy testosterone and the occurrence of GDM and found that elevated pre-pregnancy testosterone was significantly correlated with increased incidence of GDM and GDM A2. Overall, when testosterone is in Q4, the risk of developing GDM (A1+A2) increases by 76%, while the risk of developing GDM A2 increases by 126%. In terms of sensitivity analysis, we found through subgroup analysis that women age<35 had a 42% increased risk of developing GDM A2, and women BMI<24 had an 86% increased risk of developing GDM A2. Women with a BMI<24 may benefit more from pre-pregnancy testosterone testing. The testosterone levels monitored during non-pregnancy in women can provide early warning for gestational diabetes mellitus GDM and GDM A2.

### 4.2 Comparison with findings of previous studies

In previous studies, the relationship between pre-pregnancy testosterone and GDM was not yet clear. One study showed that increased testosterone level would increase the risk of non- pregnancy women with type 2 diabetes(OR = 1.37,95% CI: 1.22,1.53) (13).

Although there is no literature reporting the effect of pre-pregnancy testosterone on GDM, there have been many previous studies on the effect of early pregnancy testosterone on GDM. One studies have shown that total testosterone in early pregnancy is positively correlated with glucose concentration and diagnosis of diabetes in pregnancy (OR = 3.63, 95% CI: 1.50,8.78) (18). A national study in Finland shows that high androgen levels in early pregnancy are associated with an increased incidence rate of GDM. After adjusting for gestational age, pre-pregnancy BMI, and maternal age, the risk of developing GDM increased by 1.34 times (OR = 1.34, 95% CI: 1.04-1.73) in pregnant women with the highest third quartile testosterone levels, and by 1.32 times (OR = 1.32, 95% CI: 1.03-1.69) in those with moderate third quartile testosterone levels (19). A prospective cohort study in Hungary showed that testosterone, as the main metabolite of ovarian androgens, was significantly elevated in the GDM group (21). Another study from China found that pregnant women with GDM had significantly higher levels of testosterone during mid pregnancy compared to normal pregnant women. Compared to women with low levels of testosterone, women with elevated testosterone had a higher risk of developing GDM (OR = 2.725, 95% CI: 1.435, 5.176, P = 0.002) (22).

However, some studies have found no relationship between testosterone and diabetes. A small study on women with PCOS found that testosterone cannot predict the occurrence of GDM (23). Another study included 44 pregnant women with gestational diabetes mellitus (GDM) and 33 normal pregnant women. By collecting umbilical cord blood samples from 24–28 weeks, 30–36 weeks of pregnancy, and during and after delivery, steroid omics analysis revealed that testosterone levels in GDM pregnant women were actually lower (12). The reasons for this difference may be sample size and confounding factors. Our study was a large sample study involving 4174 pregnant women, which reduced the bias caused by sampling errors. Meanwhile, we adjusted BMI, age, TG, TC, Family history and other factors are used to avoid various exaggerated effects.

### 4.3 Interpretations

#### 4.3.1 Research implications

We believe that the relationship between testosterone and diabetes can be mainly explained from three aspects: insulin resistance, inflammation and oxidative stress, and androgen receptor (AR). Firstly, elevated testosterone levels in women are closely related to insulin resistance. Hyperandrogenism status leads to compensatory increase in insulin secretion, while inhibiting the PI3K/Akt pathway of insulin and reducing glucose uptake in peripheral tissues. Testosterone can also promote the accumulation of visceral fat in women. Inflammatory factors secreted by visceral fat, such as TNF-α and IL-6, can further aggravate insulin resistance and pancreatic β cells dysfunction, thus increasing the risk of diabetes (22, 24–26).Secondly, in terms of inflammation and oxidative stress, hyperandrogenism caused by elevated testosterone can activate monocytes and macrophages, promote the secretion of proinflammatory cytokines, inhibit the phosphorylation of insulin receptor substrate (IRS), thus interfering with insulin signal transduction, and is associated with increased mitochondrial oxidative stress and reactive oxygen species (ROS) production, further damaging pancreatic β cell function, leading to the occurrence of diabetes (27, 28). Thirdly, androgen receptor is also expressed in female pancreatic beta cells. Testosterone affects gene expression in metabolic tissues by activating androgen receptor. Abnormal androgen receptor signal in women may lead to decreased insulin sensitivity. Excessive testosterone affects the chronic activation of androgen receptor in pancreatic beta cells, which is prone to diabetes, leading to excessive insulin secretion and secondary pancreatic beta cell failure (29–32).Combined with the above mechanism research, our study further provides clinical evidence that pre-pregnancy testosterone is a potential risk factor for GDM.

#### 4.3.2 Clinical implications

Our research has significant clinical and public health implications. Our study found that elevated testosterone led to an increased incidence rate of GDM. Sensitivity subgroup analysis reveals that women with different levels of pre-pregnancy testosterone, age, and BMI have varying risks of developing GDM (A1+A2) and GDM A2, with significant differences. This difference suggests that clinicians need to pay special attention to pre-pregnancy testosterone levels to identify potential GDM patients. Women aged<35 and with a BMI<24 may benefit more from pre-pregnancy testosterone testing.

### 4.4 Strength and limitations

Our research innovatively evaluates for the first time the relationship between pre-pregnancy testosterone and GDM and concludes that elevated levels are associated with an increased risk of GDM. Our study included 771 GDM women with a large sample size, effectively avoiding sampling errors and reducing bias. In addition, we conducted a systematic and comprehensive analysis, revealing the risk of developing GDM (A1+A2) and GDM A2 among women of different ages and BMI through subgroup analysis. We adjusted the age BMI, the medical history and family history of diabetes, and the ALT, Cr, TCH, TG and other indicators were adjusted to reduce the deviation and make the results more reliable. However, our research also has certain limitations. Firstly, our study is a single center study from China and may not represent the global risk of pre-pregnancy testosterone and GDM. Future multicenter studies need to validate our results. Secondly, our study did not consider the influence of other potential factors that may affect the development of GDM, such as rapid weight gain during pregnancy. These factors are not predictable before pregnancy and thus may reduce the robustness of pre pregnancy testosterone as a biomarker for risk stratification of GDM. Third, some women only take pre pregnancy testosterone as a health examination, and they may choose other hospitals to establish records for delivery. Whether these pregnant women suffer from GDM has not been considered. Finally, we did not take into account the changes in the incidence rate of GDM caused by the COVID-19 pandemic and reduced exercise during the lockdown of the city.

### 4.5 Perspectives

Our research elucidates the relationship between pre-pregnancy testosterone and GDM, attempting to explain a new factor influencing the onset of GDM. Pre-pregnancy testosterone testing is a single, inexpensive, and mature testing method. Pre-pregnancy testosterone testing can be used as a screening strategy for GDM, especially for pregnant women with BMI<24, to detect GDM early and intervene to prevent the occurrence of other pregnancy complications. At the same time, it also provides a new direction for the joint detection of testosterone and other items before pregnancy to improve the prediction of GDM.

## Data Availability

The raw data supporting the conclusions of this article will be made available by the authors, without undue reservation.
